# Transforming Communication and Non-Technical Skills in Intermediate Care Nurses Through Ultra-Realistic Clinical Simulation: A Cross-Sectional Study

**DOI:** 10.3390/nursrep15080272

**Published:** 2025-07-29

**Authors:** Mireia Adell-Lleixà, Francesc Riba-Porquet, Laia Grau-Castell, Lidia Sarrió-Colás, Marta Ginovart-Prieto, Elisa Mulet-Aloras, Silvia Reverté-Villarroya

**Affiliations:** 1 Hospital de la Santa Creu, Jesús-Tortosa, Passeig Mossèn Valls, 1, 43590 Jesús-Tortosa, Spain; mireia.adell@saluttortosa.cat (M.A.-L.); mginovart@saluttortosa.cat (M.G.-P.); elisa.mulet@saluttortosa.cat (E.M.-A.); 2 Nursing Department, Faculty of Nursing, Universitat Rovira i Virgili, Campus Terres de l’Ebre, 43500 Tortosa, Spain; sarriol@peremata.com (L.S.-C.); silvia.reverte@urv.cat (S.R.-V.); 3 Department of Pedagogy, Universitat Rovira i Virgili, Campus Terres de l’Ebre, 43500 Tortosa, Spain; 4 Fundation Pere Mata Terres de l’Ebre, 43870 Amposta, Spain; 5 Research Group on Advanced Nursing (CARING)-161, Universitat Rovira I Virgili, 43002 Tarragona, Spain

**Keywords:** intermediate care, nursing, soft skills, high-fidelity simulation training, end-of-life care

## Abstract

**Background**: Intermediate care units face growing complexity due to aging populations and chronic illnesses. Non-technical skills such as empathy and communication are crucial for quality care. We aimed to examine the relationship between communication skills, self-efficacy, and sense of coherence among intermediate care nurses. **Methods**: We conducted an observational, cross-sectional study with 60 intermediate care nurses from three units in a Catalan hospital, Spain. Participants engaged in high-fidelity simulation using geriatric end-of-life scenarios with an ultra-realistic manikin representing a geriatric patient at the end of life. NTSs were measured using validated tools: the Health Professionals Communication Skills Scale (HP-CSS), the General Self-Efficacy Scale, and the Sense of Coherence Questionnaire (OLQ-13). Sessions followed INACSL standards, including prebriefing, simulation, and debriefing phases. **Results**: Post-simulation outcomes revealed significant gains in interpersonal competencies, with men reporting higher assertiveness (*p* = 0.015) and greater satisfaction with both the simulation experience (*p* = 0.003) and the instructor (*p* = 0.008), underscoring gender-related perceptions in immersive training. **Conclusions**: Ultra-realistic clinical simulation is effective in enhancing NTS among intermediate care nurses, contributing to improved care quality and clearer professional profiles in geriatric nursing.

## 1. Introduction

The progressive aging of the population and the increasing prevalence of chronic diseases are significantly raising the demand for healthcare services, particularly among older adults with complex health and social needs. In Catalonia (Spain), this challenge is especially pronounced in regions such as Terres de l’Ebre, where over-aging and dependency rates are among the highest in the region (over-aging index: 19.3% vs. 17.1% in Catalonia; dependency index: 58.1%, reaching 70.9% in Terra Alta) [1,2]. This demographic and epidemiological shift is placing sustained pressure on healthcare systems and calls for the development of adaptable, efficient, and patient-centered care models.

Within the evolving landscape of healthcare, intermediate care (IC) has become a crucial part of the care continuum in Catalonia. It brings together a variety of services focused on helping people recover their functional abilities after an acute illness, reducing unnecessary hospital admissions, enabling patients to leave the hospital sooner, and supporting those with chronic or complex conditions to live more independently [3]. These services take place in various settings such as hospitals, long-term care facilities, and patients’ homes and are provided by interdisciplinary teams who work under a holistic biopsychosocial approach [4].

In the Terres de l’Ebre region, the Hospital de la Santa Creu in Jesús functions as the regional reference center for intermediate care, offering specialized units tailored to meet the varied needs of this population. Post-acute care units primarily focus on multidisciplinary interventions to enhance functional recovery after acute medical, surgical, or traumatic events, with particular attention paid to patients living with chronic conditions. Palliative care units emphasize comfort and symptom management for patients facing advanced cancer or other terminal illnesses while also addressing the emotional and spiritual support needs of both patients and their families. Additionally, long-term care units cater to older adults with prolonged chronic illnesses that result in functional dependency, including those suffering from advanced dementia and other cognitive impairments [4].

Together, these services embody a comprehensive and patient-centered approach aimed at optimizing health outcomes and enhancing quality of life throughout the care continuum in Catalonia.

As care becomes more complex, especially in intermediate care settings, nurses increasingly need to develop non-technical skills, also called soft skills. These skills include communication, leadership, teamwork, ethical reasoning, and decision-making. Non-technical skills are cognitive, social, and personal resources that help professionals perform tasks safely and effectively in complex situations [5]. They involve abilities such as decision-making, teamwork, leadership, situational awareness, communication, and task management. These skills complement technical abilities and are essential for effective performance, especially in high-risk, team-based healthcare environments [6,7].

To meet these training needs, clinical simulation has emerged as an innovative educational methodology. It enables learners to engage in realistic clinical scenarios within a safe, controlled environment, fostering critical thinking, self-confidence, and reflective practice without putting patients at risk. High-fidelity and ultra-realistic simulations, including the use of standardized patients or trained actors, enhance emotional engagement and improve knowledge transfer to real-world settings [8]. The simulation process typically follows a structured framework consisting of prebriefing, scenario enactment, and debriefing, aligning with best practices in nursing education.

Evidence suggests that structured simulation-based training not only strengthens technical and clinical competencies but also significantly enhances NTSs, including interpersonal communication, collaboration, and clinical decision-making [5]. This is particularly relevant in IC units, where effective team dynamics and patient-centered communication are critical to managing complex care processes.

In response to these challenges and the evolving demands of contemporary healthcare, the present study aims to integrate hyper-realistic clinical simulation as a training strategy to develop non-technical skills among nurses working in intermediate care settings.

### Hypothesis and Objective

We hypothesized that intermediate care nurses with practical communication skills, self-efficacy, and sense of coherence would perform better in simulated clinical scenarios.

This study aimed to assess the relationship between communication skills, self-efficacy, and sense of coherence in intermediate care nurses and how these factors influence performance in simulated clinical scenarios involving a high-fidelity, ultra-realistic manikin representing a geriatric patient at the end of life.

## 2. Materials and Methods

### 2.1. Design

A descriptive, observational, and cross-sectional study was carried out from 16 January 2024, to 2 October 2024 to explore the development of non-technical skills (NTSs), particularly interpersonal competencies, among nurses working in intermediate care (IC) units through the use of ultra-realistic clinical simulation. This study adheres to the STROBE guidelines for reporting cross-sectional studies [9].

### 2.2. Participants and Study Settings

A non-probabilistic, intentional sampling method was used. The study population consisted of 60 nurses employed at the intermediate care units of the Hospital de la Santa Creu (Jesús-Tortosa, Catalonia, Spain). Subacute care units provide comprehensive attention to individuals with chronic, advanced conditions who experience acute exacerbations, requiring moderate levels of clinical intervention. The goal is clinical stabilization and functional rehabilitation to enable early discharge with maximum autonomy.

The inclusion criteria were (1) registered nurses working in IC units and who voluntarily agreed to participate, (2) nurses who attended all the clinical simulation sessions in zone 3 [10], (3) those who adequately completed all the evaluation questionnaires, and (4) those who gave informed consent. The exclusion criteria were (1) nurses working in long-term care facilities, nephrology, or outpatient services (e.g., primary care support teams, hospital-based support teams, dependency assessment units, or outpatient clinics). In the present study, clinical scenarios were conducted in a high-fidelity simulation environment, following the standards established by INACSL [11]. Two simulated clinical scenarios focused on the care of a patient at the end of life were designed, using an ultra-realistic mannequin, in line with INACSL design recommendations [11]. These scenarios were developed using the GRISANE methodology, specifically adapted for simulating non-technical skills [12].

The intervention took place at the Territorial Innovation and Simulation Centre (CISTE) and was facilitated by an instructor with experience in conducting clinical simulations in intermediate care settings. The process began with a prebriefing, during which a psychologically safe environment was established, utilizing group dynamics and guidelines based on best practices. This was followed by an introduction to the simulated scenario, explaining the roles of the participants, the environment, and the session objectives [13,14].

During the simulation, participants voluntarily took part in the simulated case, with four individuals required to conduct each simulation. Group members who were not actively involved in the scenario as confederate actors or healthcare professionals observed from an adjacent room with one-way glass, allowing them to watch the simulation without feeling observed themselves, thus promoting observational learning. At the end of each scenario, a structured debriefing session was conducted using the GAS method (gather, analyze, and summarize) alongside the delta-plus technique. This approach facilitated reflection on participants’ performance, with a particular focus on non-technical skills such as communication, empathy, and decision-making [15].

### 2.3. Variables and Measurement Instruments:

This study took into account the following variables:
  1. Sociodemographic variables: age and sex (male, female, non-binary).
  2. Professional variables: years of professional experience, specific IC unit, years of experience in intermediate care, prior training in NTSs, other relevant training, work shifts, specialty in geriatrics, and satisfaction with the simulation methodology.
  3. Non-technical skills variables: communication abilities, empathy, respect, social skills, and self-efficacy.
  4. Additional psychological variable: self-efficacy and sense of coherence.
Data were collected through the following validated instruments (Table 1):

Sociodemographic and professional data were collected through a self-administered ad hoc questionnaire designed using Microsoft Forms. Data related to NTSs were collected using the following instruments:

(a) Health Professionals’ Communication Skills Scale (EHC-PS) [16,17]. This is a self-administered instrument composed of 18 items rated on a 6-point Likert scale. It includes four dimensions:
  - Informative communication (6 items: 5, 8, 9, 14, 17, 18), which reflects the ability of health professionals to obtain and provide clinical information. Scores range from 6 to 36.
  - Empathy (5 items: 2, 4, 6, 11, 12), which evaluates the capacity to understand patients’ emotions and demonstrate this understanding in a clinical relationship, including active listening and empathic responses. Scores range from 5 to 30.
  - Respect (3 items: 1, 3, 15), assessing the respectful behavior shown by professionals in their interactions with patients. Scores range from 3 to 18.
  - Social skills (4 items: 7, 10, 13, 16), which measure assertiveness and the ability to exhibit socially appropriate behavior during clinical interactions. Scores range from 4 to 24.
(b) The General Self-Efficacy Scale, consisting of 10 items that evaluate the individual’s belief in their ability to effectively manage a wide range of stressors in everyday life. Scores range from 10 to 40 [18].
(c) Sense of Coherence Questionnaire (OLQ-13) by Antonovsky [19]. The short version (OLQ-13) was used, which includes 13 items rated on a 7-point Likert scale and assesses three components: comprehensibility (items 2, 6, 8, 9, 11), manageability (items 3, 5, 10, 13), and meaningfulness (items 1, 4, 7, 12). Total scores range from 13 to 91.
(d) Satisfaction Questionnaire—CISTE. The satisfaction survey developed by CISTE was used to evaluate participants’ opinions regarding the training activity, instructors, and facilities. The questionnaire includes 16 items rated on a 10-point Likert scale (1 = not at all satisfied; 10 = completely satisfied).

### 2.4. Statistical Methods

A descriptive analysis was performed for all study variables. Qualitative variables were summarized using absolute frequencies and corresponding percentages. Quantitative variables were described using either the mean and standard deviation or the median and interquartile range (IQR), depending on the distribution of the data. Normality was assessed using the Kolmogorov–Smirnov test. Differences between pre- and post-simulation assessments were analyzed using the Mann–Whitney U or Kruskal–Wallis test depending on whether it was a comparison of means between 2 samples or among k samples, as the study design involved independent groups. All statistical analyses were conducted using SPSS version 27.0 (IBM Corp., Armonk, NY, USA), licensed to the Universitat Rovira Virgili (URV). The level of statistical significance was set at *p* < 0.05.

### 2.5. Ethical Considerations

This study was carried out in accordance with the ethical principles set forth in the Declaration of Helsinki [20]. Participation was entirely voluntary, and all nursing professionals involved were informed that declining or withdrawing from the study at any time would have no negative academic or professional consequences.

To preserve data confidentiality and participant anonymity, each questionnaire was coded using an alphanumeric identifier, and identifying information was collected from participants. It is important to note that anonymity applies solely to the data gathered, not to the individuals themselves. All participants provided informed consent prior to their involvement in the study. The research received a favorable opinion from the Ethics Committee for Research with Medicines of the Pere Virgili Health Research Institute (Institut d’Investigació Sanitària Pere i Virgili), with approval code CEIM: 143/2023, on 27 July 2023.

## 3. Results

### 3.1. Description of the Participants

A total of 62 nurses were initially recruited for the study. However, two cases (3.2%) were excluded due to incomplete responses in the post-intervention questionnaires, resulting in a final sample of 60 participants. The majority were women (83%), with a mean age of 38 years (SD = 12.11). Participants had an average of 16 years of professional experience (SD = 12.10), and 50% reported having received prior training in clinical simulation methodology.

### 3.2. Results of Assessment Tool

The analysis explored the association between years of professional experience and the development of non-technical skills, specifically in the dimensions of Informative Communication, Empathy, Respect, and Assertiveness. Participants were categorized into five groups according to their years of experience: less than 1 year, less than 5 years, 5–10 years, 11–20 years, and more than 20 years. In general, the median scores in Informative Communication, Empathy, and Respect remained relatively stable across all experience groups, with no statistically significant differences (*p* > 0.05 in all three dimensions). However, a statistically significant difference was observed in the Assertiveness dimension (*p* = 0.002), where nurses with 5–10 years of experience reported the highest median score (M = 5, SD = 0.68), suggesting greater self-perceived assertiveness. In contrast, those with less than 5 years and more than 10 years of experience showed lower scores, especially participants with more than 20 years of experience (M = 4, SD = 0.57) (Table 2).

Table 3 shows the NTS scores in four dimensions—informational communication, empathy, respect, and assertiveness—distributed among the participants by gender (women and men). The table shows the median (M) and standard deviation (SD) for each dimension for women (n = 50) and men (n = 10), together with the *p*-values for each comparison between the two groups, e.g., informative communication: women M = 4.43 (SD = 0.36), men M = 4.74 (SD = 0.36), and *p*-value = 0.042. The results indicate that there are SI significant differences in informative communication between women and men. For empathy—with women M = 4.54 (SD = 0.43), men M = 4.71 (SD = 0.43), and *p*-value = 0.322—the *p*-value being above 0.05 indicates that there is no statistically significant difference in empathy scores between women and men. Although men report a slightly higher median score, the difference is not statistically significant. For respect, women M = 4.68 (SD = 0.44), men M = 4.52 (SD = 0.63), and *p*-value = 0.042. No significant differences were found between women and men regarding respect, as evidenced by a *p*-value of 0.042. Both groups show comparable scores on this dimension. For assertiveness, women M = 3.97 (SD = 0.58), men M = 4.57 (SD = 0.55), and *p*-value = 0.015. This dimension shows a significant difference, with *p*-value = 0.015, indicating that men scored significantly higher on assertiveness than women.

Table 4: Satisfaction levels were evaluated across four dimensions: simulation, manikin, expert instructor, and CISTE space (Table 3). The median satisfaction score for the simulation was 8.61 (SD = 1.19), with scores ranging from 6 to 10. The first quartile (Q1) was 8, and the third quartile (Q3) was 9.75. The *p*-value for the comparison based on years of professional experience was 0.064, indicating no significant differences in satisfaction with the simulation based on experience. For the manikin, the median satisfaction score was 9.39 (SD = 0.78), with a range from 7 to 10. The Q1 score was 9, and the Q3 score was 10. The *p*-value for the comparison was 0.183, suggesting no significant differences in satisfaction with the manikin based on years of professional experience. The median satisfaction score for the expert instructor was 9.30 (SD = 0.83), with scores ranging from 7 to 10. The Q1 score was 8.5, and the Q3 score was 10. The *p*-value was 0.341, showing no significant difference in satisfaction based on years of professional experience. The CISTE space received a median satisfaction score of 9.48 (SD = 0.73), with a range from 7 to 10. The Q1 score was 9, and the Q3 score was 10. The *p*-value was 0.195, indicating no significant differences in satisfaction with the CISTE space based on years of professional experience.

Table 5 presents the satisfaction scores for different aspects of the simulation experience, comparing women and men. The scores were measured using the median and standard deviation (SD), with statistical significance tested using *p*-values. Regarding the simulation, women reported a median satisfaction score of 8.33 (SD = 1.12), while men reported a score of 10. The difference between genders was statistically significant, with a *p*-value of 0.003, indicating that men reported significantly higher satisfaction with the simulation experience compared to women. With respect to the manikin, both women (M = 9.50, SD = 0.81) and men (median = 10) reported high satisfaction with the manikin, but the difference between genders was not statistically significant (*p*-value = 0.202). On the other hand, for the expert instructor, women gave the instructor M = 9.50 (SD = 0.97), while men rated the instructor a perfect 10. The *p*-value = 0.008 indicates that there was a significant difference between the genders, with men expressing higher satisfaction with the expert instructor than women.

Finally, for CISTE space, women reported a median satisfaction score of 9.67 (SD = 0.77), while men rated the CISTE space a perfect 10. Although men reported a slightly higher median score, the difference was not statistically significant (*p* = 0.068), suggesting comparable satisfaction levels between groups.

## 4. Discussion

This study explored the influence of ultra-realistic clinical simulation on the development of NTSs in nurses working in intermediate care settings. The results showed high post-simulation scores across all four NTS dimensions (informative communication, empathy, respect, and assertiveness), suggesting that the simulated experience was effective in promoting key interpersonal and emotional competencies [21].

One of the most remarkable findings was the consistency in outcomes regardless of years of professional experience. Both early-career and seasoned nurses reported similar levels of improvement, highlighting that ultra-realistic simulation may serve as a valuable and inclusive training strategy [22]. This supports the idea that non-technical skills, unlike procedural skills, are not exclusively shaped by clinical seniority but can be effectively enhanced through immersive experiential learning. Interestingly, the results suggest that mid-career professionals (5–10 years of experience) may perceive themselves as more assertive in clinical interactions, possibly due to the balance between accumulated experience and active engagement in ongoing professional development [23]. This nuance adds further insight into the evolving trajectory of interpersonal competence over the course of a nursing career [24].

Gender differences in competencies such as assertiveness, often influenced by social norms, cultural factors, and gender stereotypes, may represent a key opportunity for professional development through clinical simulation. In this study, male participants tended to report higher levels of assertiveness; however, this finding should be interpreted with caution, considering potential social desirability bias and cultural expectations that shape how men and women respond to questionnaires and express their experiences [24,25].

These dynamics need not be obstacles but rather enriching elements in clinical simulation, which can offer a safe space to normalize and promote assertive communication across genders. By recreating ultra-realistic clinical scenarios, simulation challenges stereotypes and fosters critical reflection and the development of essential non-technical skills that support equality and inclusion in healthcare.

Therefore, it is recommended that future simulation program designs and evaluations explicitly incorporate a gender perspective to ensure more accurate interpretation of results and to support more equitable professional development.

Although the simulation instructor was female, male participants reported slightly higher satisfaction with her skills and teaching approach. This difference may reflect varying expectations and perceptions influenced by the participants’ gender, as well as a potential alignment between the instructor’s communication style and the learning preferences of male participants [23]. The current literature does not identify the instructor’s gender as a determining factor in participant satisfaction, instead emphasizing the importance of didactic competence, empathy, and the ability to create a safe and motivating learning environment. The observed difference might also stem from group-specific dynamics and individual variability, highlighting the need for qualitative studies to explore these perceptions in greater depth.

The relationship between communication-related competencies and both self-efficacy and sense of coherence was also noteworthy [26]. Participants who scored higher in informative communication and empathy also tended to exhibit greater self-efficacy and a stronger sense of coherence [26,27]. This suggests that effective interpersonal communication is not only a skill but also a reflection of the individual’s psychological resilience and ability to find meaning and manage complexity in clinical practice. A higher sense of coherence—particularly in the dimensions of manageability and meaningfulness—appears to be aligned with an empathetic and respectful approach to patient care.

These findings reinforce the idea that communication skills and emotional intelligence are deeply interconnected to a nurse’s perceived capacity to navigate challenging scenarios and maintain psychological wellbeing. This is especially relevant in the context of end-of-life care, where both technical proficiency and emotional presence are essential [28,29].

From a practical perspective, these findings highlight the clear benefits of incorporating ultra-realistic simulation into the ongoing professional development of nurses. Training programs that include the development of NTSs through simulation can address important gaps often overlooked in traditional education [30,31], especially in complex care settings such as intermediate care units [32].

Moreover, satisfaction scores were consistently high, particularly with regard to the quality of the teaching staff and the realism of the simulation environment. Participants valued the immersive experience, reporting that it provided a psychologically safe and professionally enriching space [33,34]. This highlights the importance of not only what is taught but how it is taught—simulations that integrate fidelity, structured debriefing, and emotionally charged scenarios can foster significant learning outcomes and professional growth.

This approach reinforces the notion that ultra-realistic simulation training not only enhances technical and interpersonal skills but also cultivates an educational environment that promotes confidence, resilience, and psychological wellbeing among healthcare professionals. Integrating this methodology into institutional training plans could represent a major advancement in preparing nurses to face the complexities of contemporary clinical practice [35].

The present study has several limitations that should be acknowledged. First, the sample consisted exclusively of nurses working in intermediate care units within a specific geographical region, and the sampling procedure was non-probabilistic. This may limit the generalizability of the findings to other professional contexts or healthcare settings. Second, although the sample included both male and female participants, only 11 identified as male. This small number may reduce the representativeness of gender-based comparisons. Third, the study employed a cross-sectional post-intervention design without the inclusion of a control group. As a result, it is not possible to attribute the observed changes in non-technical skills solely to the simulation-based intervention, which limits the ability to draw causal inferences. Another limitation concerns the use of self-reported measures for data collection. Although commonly used in research, self-reports are subject to social desirability bias and may not always accurately reflect actual behavior.

Lastly, the evaluation of outcomes was conducted immediately after the simulation session. Therefore, the study does not provide information on the long-term retention of non-technical skills or the transfer of these competencies to clinical practice. Further research with follow-up assessments is needed to evaluate the sustained impact of ultra-realistic simulation training [36,37].

Nevertheless, this study offers relevant evidence on the educational value of simulation-based training for strengthening essential nursing competencies. The integration of non-technical skills training within realistic clinical scenarios, facilitated through rigorous methodologies such as GRISANE, represents a powerful and innovative approach for professional development in nursing.

## 5. Conclusions

This study highlights the educational value of ultra-realistic clinical simulation as an effective strategy for developing non-technical skills among nurses working in intermediate care settings. The findings indicate that immersive simulation scenarios focusing on end-of-life care can strengthen essential communication competencies such as empathy, assertiveness, and respectful interactions. These skills are fundamental to the delivery of high-quality, compassionate, and person-centered care.

Improvements were observed across professionals with different levels of experience, which reinforces the inclusive and adaptable nature of simulation-based training. The gender-related differences found in assertiveness, likely influenced by sociocultural factors, point to the importance of integrating a gender-sensitive perspective into the design and evaluation of simulation programs. Simulation provides a safe and structured environment in which participants can reflect on and challenge communication patterns, supporting the development of more equitable and confident professional practices.

The positive correlation between communication skills, self-efficacy, and sense of coherence suggests a close relationship between emotional regulation, professional confidence, and the capacity to find meaning in clinical work. These results support the integration of simulation-based learning into ongoing professional development to promote comprehensive nursing competencies.

Although this study has certain limitations, such as sample size and reliance on self-reported data, it offers meaningful evidence of the pedagogical potential of ultra-realistic simulation. Future research should aim to evaluate the long-term retention of skills, assess their transferability to clinical practice, and examine their impact on patient outcomes.

## Figures and Tables

**Table 1:** Summary of instruments used.

Instrument	Number of Items	Scale Type	Dimensions	Score Range	Main Purpose
(a) Health Professionals’ Communication Skills Scale (EHC-PS)	18	6-point Likert	Informative Communication, Empathy, Respect, Social Skills	6–36, 5–30, 3–18, 4–24	To assess communication and social skills
(b) General Self-Efficacy Scale	10	Single scale	—	10–40	To measure self-efficacy in stress management
(c) Sense of Coherence Questionnaire (OLQ-13)	13	7-point Likert	Comprehensibility, Manageability, Meaningfulness	13–91	To assess the sense of coherence
(d) Satisfaction Questionnaire—CISTE	16	10-point Likert	—	1–10	To evaluate training activity satisfaction

**Table 2:** Relationship between professional experience and non-technical skills (NTSs).

Years of Professional Experience	Informative Communication Median (SD)	*p*-Value	Empathy Median (SD)	*p*-Value	Respect Median (SD)	*p*-Value	Assertiveness Median (SD)	*p*-Value
<1 year	5.00 (0.38)		4.80 (0.53)		5.00 (0.38)		4.50 (0.63)	
<5 years	4.67 (0.42)		4.60 (0.53)		4.67 (0.42)		4.25 (0.38)	
5–10 years	5.00 (0.38)		5.00 (0.35)		5.00 (0.38)		5.00 (0.68)	
11–20 years	5.00 (0.47)		4.60 (0.49)		5.00 (0.47)		4.00 (0.56)	
>20 years	4.67 (0.32)		4.60 (0.40)		4.67 (0.32)		4.00 (0.57)	
Statistical significance		0.125		0.191		0.125		0.002 *

Non-parametric comparisons between groups based on years of professional experience and communication domains were performed using the Kruskal–Wallis test. Statistical significance was set at *p* < 0.05 (*).

**Table 3:** Relationship between the scores for non-technical skills (NTSs) and gender (women and men).

Sex	Informative Communication Median (SD)	Empathy Median (SD)	Respect Median (SD)	Assertiveness Median (SD)
Women (n = 45)	4.43 (0.36)	4.54 (0.43)	4.68 (0.44)	3.97 (0.58)
Men (n = 11)	4.74 (0.36)	4.71 (0.43)	4.52 (0.63)	4.57 (0.55)
*p*-value	0.042	0.322	0.441	0.015 *

Comparisons between men and women across communication domains were analyzed using the Mann–Whitney U test; statistical significance was set at *p* < 0.05 (*).

**Table 4:** Satisfaction scores for simulation, manikin, expert instructor, and CISTE space based on years of professional experience.

Satisfaction Item	Median (SD)	Min	Max	Q1 (25%)	Q3 (75%)	*p*-Value (Years of Experience)
Simulation	8.61 (1.19)	6	10	8	9.75	0.064
Manikin	9.39 (0.78)	7	10	9	10	0.183
Expert Instructor	9.30 (0.83)	7	10	8.5	10	0.341
CISTE Space	9.48 (0.73)	7	10	9	10	0.195

Statistical differences by years of professional experience were analyzed using the Kruskal–Wallis test. Statistical significance was set at *p* < 0.05.

**Table 5:** Satisfaction scores by gender for simulation, manikin, expert instructor, and CISTE space.

Satisfaction Item	Woman (n = 37)	Man (n = 11)	*p*-Value
Simulation	8.40 (1.18)	9.71 (0.40)	0.003 *
Manikin	9.32 (0.81)	9.71 (0.57)	0.202
Expert Instructor	9.16 (0.84)	10 (0.00)	0.008 *
CISTE Space	9.41 (0.77)	9.90 (0.25)	0.068

Group differences were analyzed using the Mann–Whitney U test. Statistical significance was set at *p* < 0.05 (*).

## Data Availability

The original contributions presented in this study are included in the article. Further inquiries can be directed to the corresponding authors.

## Public Involvement Statement

There was no public involvement in any aspect of this research.

## Guidelines and Standards Statement

This manuscript was drafted against the STROBE guidelines (Strengthening the Reporting of Observational Studies in Epidemiology).

## Abbreviations

The following abbreviations are used in this manuscript:

CEIM	Ethics Committee for Research with Medicine
CISTE	Territorial Innovation and Simulation Centre
HP-CSS	Health Professionals Communication Skills Scale
IC	Intermediate Care
IQR	Median Interquartile Range
NTS	Non-Technical Skills
OLQ-13	Sense of Coherence Questionnaire
URV	Universitat Rovira i Virgili
